# Integrin αM promotes macrophage alternative M2 polarization in hyperuricemia‐related chronic kidney disease

**DOI:** 10.1002/mco2.580

**Published:** 2024-06-22

**Authors:** Jing Liu, Fan Guo, Xiaoting Chen, Ping Fu, Liang Ma

**Affiliations:** ^1^ Division of Nephrology, Institute of Kidney Diseases West China Hospital of Sichuan University Chengdu China; ^2^ Animal Experimental Center West China Hospital of Sichuan University Chengdu China

**Keywords:** chronic kidney disease, hyperuricemia, integrin αM, macrophage M2 polarization

## Abstract

Hyperuricemia is an essential risk factor in chronic kidney disease (CKD), while urate‐lowering therapy to prevent or delay CKD is controversial. Alternatively activated macrophages in response to local microenvironment play diverse roles in kidney diseases. Here, we aim to investigate whether and how macrophage integrin αM (ITGAM) contributes to hyperuricemia‐related CKD. In vivo, we explored dynamic characteristics of renal tissue in hyperuricemia‐related CKD mice. By incorporating transcriptomics and phosphoproteomics data, we analyzed gene expression profile, hub genes and potential pathways. In vitro, we validated bioinformatic findings under different conditions with interventions corresponding to core nodes. We found that hyperuricemia‐related CKD was characterized by elevated serum uric acid levels, impaired renal function, activation of macrophage alternative (M2) polarization, and kidney fibrosis. Integrated bioinformatic analyses revealed *Itgam* as the potential core gene, which was associated with focal adhesion signaling. Notably, we confirmed the upregulated expression of macrophage ITGAM, activated pathway, and macrophage M2 polarization in injured kidneys. In vitro, through silencing *Itgam*, inhibiting p‐FAK or p‐AKT1 phosphorylation, and concurrent inhibiting of p‐FAK while activating p‐AKT1 all contributed to the modulation of macrophage M2 polarization. Our results indicated targeting macrophage ITGAM might be a promising therapeutic approach for preventing CKD.

## Graphical abstract

Schematic diagram of macrophage ITGAM and related mechanisms in hyperuricemia‐related chronic kidney disease in mice. Through the integration and analysis of transcriptomic and phosphoproteomic data from renal tissue stimulated by hyperuricemia, we identified ITGAM as a central node. ITGAM on macrophages activates FAK/AKT/GSK‐3β, resulting in the stabilization of β‐catenin, and promotion of M2 polarization and kidney fibrosis in hyperuricemic CKD mice. The findings were confirmed through both in vivo and in vitro experiments by manipulating *Itgam*, p‐FAK, and p‐AKT1.

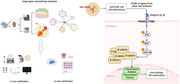

## 1 INTRODUCTION

Hyperuricemia prevalence has been rising in recent years, attributable in part to shifts toward unhealthy dietary patterns and lifestyles.^1^, ^2^ The kidneys in healthy individuals are responsible for the excretion of two‐third uric acid (UA), thus playing an essential role in the pathogenesis and progression of chronic kidney disease (CKD). Kidney damage related to hyperuricemia typically manifests as chronic interstitial nephritis, the formation of urate crystals or stones, and subsequent kidney fibrosis.^3^, ^4^ As a modifiable metabolite, UA represents a viable therapeutic target for mitigating kidney damage induced by hyperuricemia. But in the setting of CKD, it is controversial whether UA‐lowering is an effective strategy to prevent or delay CKD progression.^5^ Investigations into therapeutic targets that participate in hyperuricemia‐related CKD are of great clinical significance.

Integrin constitutes the largest family of cell adhesion molecules and is involved in kidney development and diseases.^6^, ^7^ Renal fibrosis could be induced by integrins through cell–matrix or cell–cell interactions.^8^ Integrins are αβ heterodimeric transmembrane glycoproteins and mainly divided into integrin β1, β2, and β3 families according to β subunits.^9^ As transmembrane receptors, integrins participate in cell proliferation, survival and migration, differentiation, and matrix homeostasis.^8^ Due to lack of enzymatic activity, integrins need to bind adaptor proteins for intracellular signal propagation, such as focal adhesion kinase (FAK), a key tyrosine kinase of intracellular signaling binding to a number of downstream molecules.^10^

αMβ2, also known as macrophage antigen 1 (Mac‐1), is the predominant leukocyte‐specific β2 integrin abundantly expressed in monocytes/macrophages and dendritic cells.^11^ The αMI‐domain within αMβ2 mediates ligand binding and is responsible for substrate specificity, thus integrin αM (ITGAM) mainly determined diverse functions of Mac‐1.^12^ Previous studies reported that ITGAM overexpression was associated with macrophage infiltration and renal fibrosis.^13^, ^14^ Macrophages accumulate in injured kidneys and present as polarized M1 or M2 phenotype for proinflammatory or profibrotic functions, respectively.^15^, ^16^ Macrophage alternative (M2) polarization is considered as an essential feature of fibrosis.^17^ However, it is not well elucidated whether ITGAM regulated macrophage M2 polarization in renal fibrosis and signaling pathways involved.

Here, integrin ITGAM is reported as the hub gene promoting macrophage M2 polarization in hyperuricemia‐related CKD. Through comprehensive bioinformatic analysis, we have confirmed that ITGAM contributes to the development of kidney disease by modulating the FAK/AKT1/GSK‐3β signaling pathway. Our findings shed light on the molecular mechanism underlying kidney fibrosis in hyperuricemia‐related CKD, highlighting the significance of ITGAM expression, and signaling as potential therapeutic targets for the prevention and delay of CKD progression.

## 2 RESULTS

### 2.1 Progressive renal function decline and kidney fibrosis in mice wth hyperuricemia‐related CKD

**FIGURE 1:**
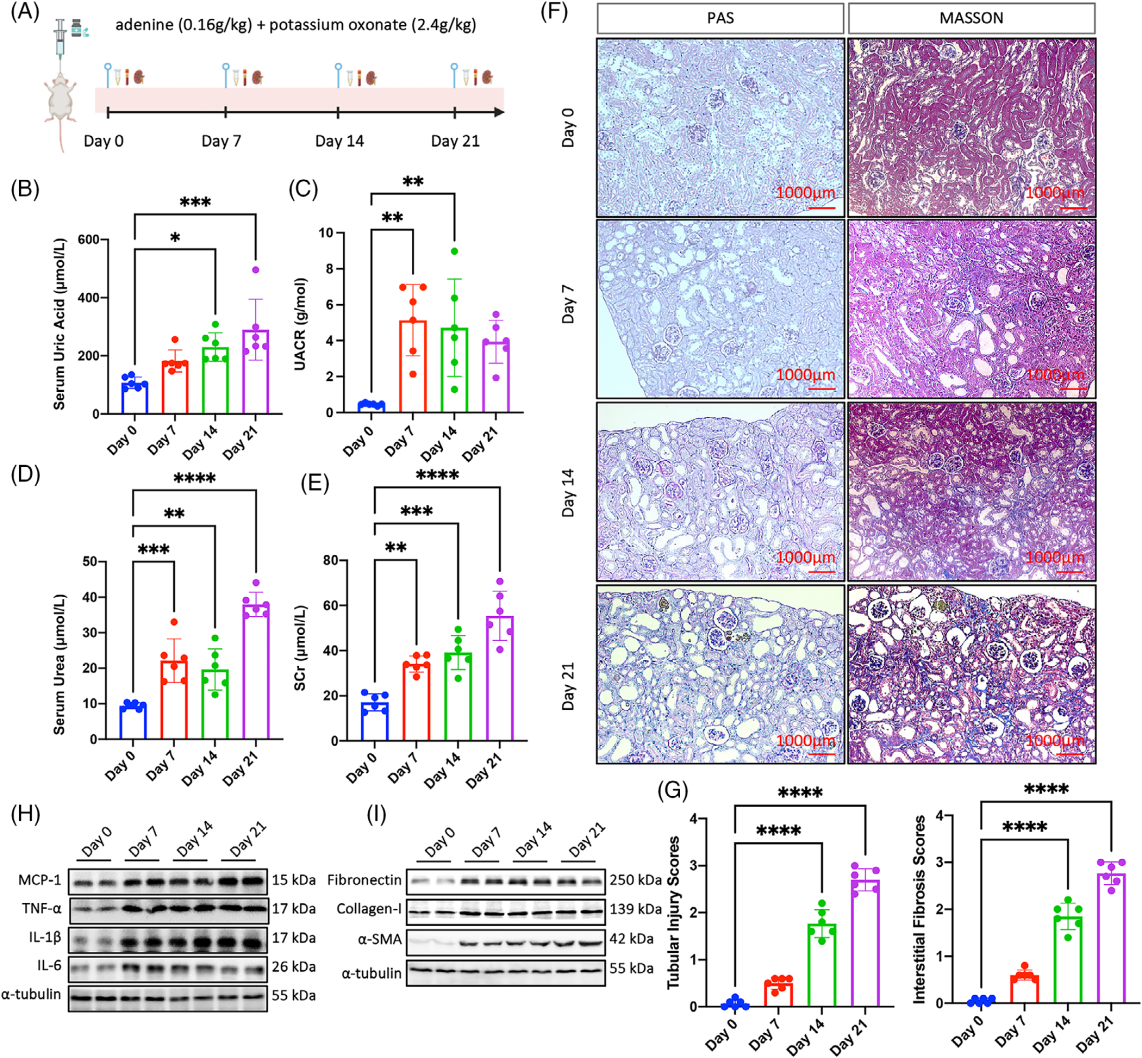
Progressive renal dysfunction and kidney fibrosis in hyperuricemia‐related CKD mice. (A) Brief instruction on model establishment and sample collection. (B) Quantification of serum uric acid from day 0 to day 21. (C) Quantification of urine albumin‐to‐creatinine ratio (UACR) from day 0 to day 21. (D) Quantification of serum urea from day 0 to day 21. (E) Quantification of serum creatinine (SCr) from day 0 to day 21 (F) Representative images of periodic acid‐Schiff (PAS) and Masson trichrome staining, indicating progressive tubular atrophy, glomerulosclerosis, and interstitial collagen deposition. (G) Quantification and comparison of tubular injuries and interstitial fibrosis across time points. (H) Protein expression changes in inflammatory indicators (MCP‐1, TNF‐α, IL‐1β, and IL‐6) from day 0 to day 21 in renal tissue. (I) Protein expression changes of profibrotic indicators (fibronectin, collagen‐1, and α‐SMA) from day 0 to day 21 in renal tissue. Each experiment had a sample size of *n* = 6; ****p < 0.0001, ***p < 0.001, **p < 0.01, *p < 0.05.

To investigate the phenotypic dynamic changes of hyperuricemia‐related CKD, we conducted animal model in mice and performed biochemical and renal tissue analyses. From day 0 to day 21 under gavage feeding of adenine and potassium oxonate, we set several time points and described dynamic characteristics of blood, urine, and kidney samples (Figure 1A). Serum UA kept rising and reached peak at day 21 (Figure 1B). Similar trend to UA was observed in renal function measurements. Urine albumin‐to‐creatinine ratio (UACR) rose rapidly and became five fold level at day 7 compared with day 0, and then slowly declined but still at high level at days 14 and 21 (Figure 1C). Serum urea and serum creatinine (SCr) went up quickly from day 0 to day 7, kept stable at high level at day 14, and then sharply increased to three and four times of baseline level respectively (Figures 1D and E). Histologic and quantitative changes observed in periodic acid‐Schiff (PAS)‐staining and Trichrome‐staining sections indicated progressive tubular and glomerular injuries, as well as increased collagen deposition in the tubular interstitial and glomerular mesangial compartments, respectively (Figures 1F and G). We further observed progressive inflammatory cell recruitment indicated by chemokine MCP‐1, proinflammatory cytokine TNF‐α, IL‐6, IL‐1β (Figure 1H), as well as profibrotic marker fibronectin, collagen‐I, and α‐SMA (Figure 1I). The dynamic evolution of hyperuricemia‐related CKD is characterized by a progressive increase in blood UA levels and a decline in renal function. The renal tissue phenotype predominantly exhibits persistent inflammation and progressive fibrosis.

### 2.2 *Itgam* is the hub gene potentially activating focal adhesion pathway in mice with hyperuricemia‐related CKD

**FIGURE 2:**
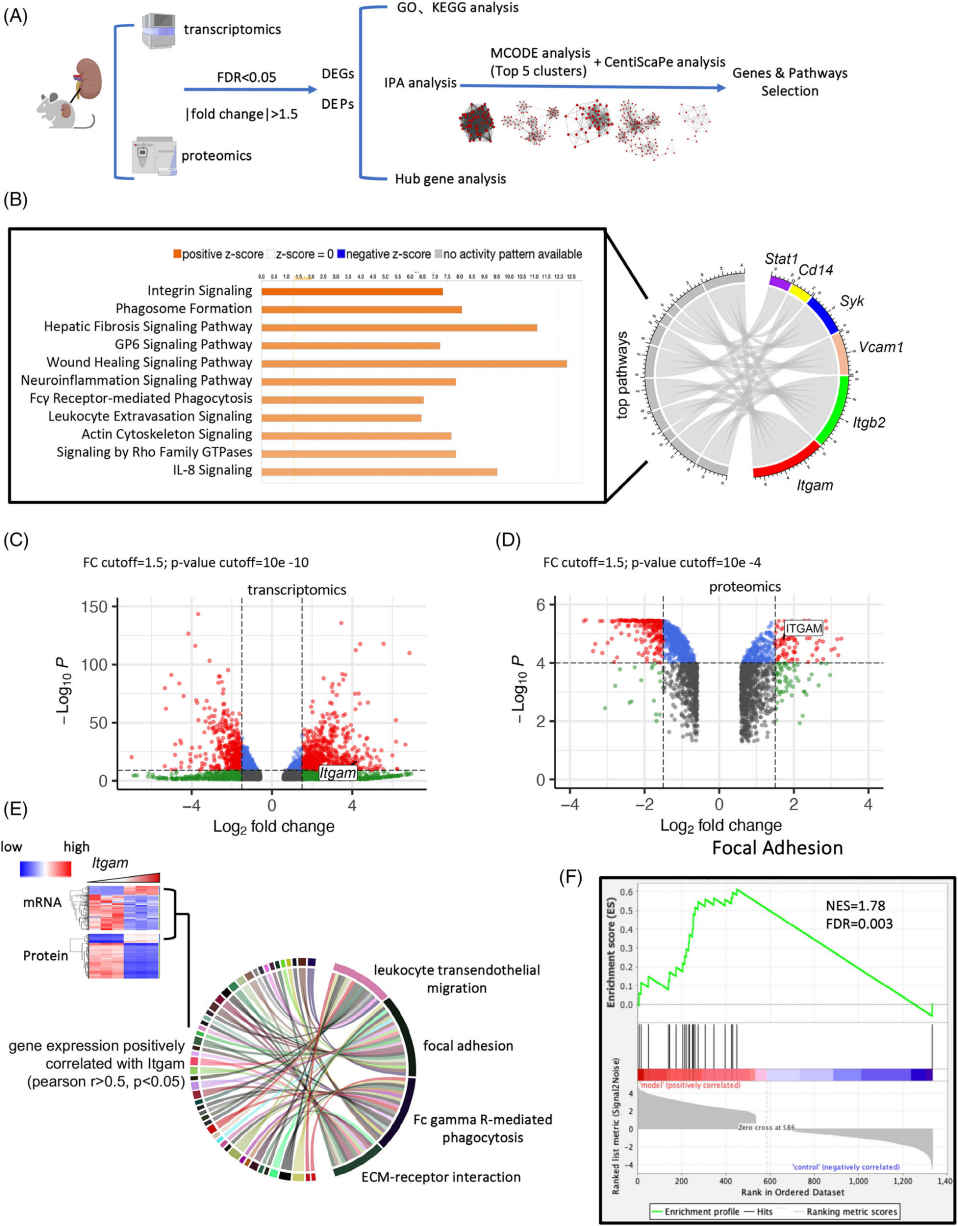
Bioinformatic analyses revealed *Itgam* as the hub gene and downstream focal adhesion pathway. (A) Sketch map of how we incorporated mRNA and protein sequencing data to select core genes and pathways related. In brief, we found out 1337 DEGs both at mRNA and protein levels and further adopted hub gene analyses (MCODE followed by CentiScape in Cytoscape) to acquire simplified 49 genes. (B) There are 6 of 49 hub genes corresponding to top canonical pathways conducted by IPA. Itgam and Itgb2, as two heterodimers of Mac‐1, were top‐ranked. (C and D) Itgam on day 21, at mRNA and protein level, was upregulated to 19 and 3.3 times of day 0. (E) KEGG results based on genes significantly positively correlated with Itgam at both mRNA and protein levels; (F) Top1 GSEA analysis of DEGs and DEPs is focal adhesion pathway.

To extract essential molecules from a data‐driven perspective, we conducted mRNA and protein sequencing on mouse kidneys and performed comprehensive integrated analyses. Differentially expressed genes (DEGs) and differentially expressed proteins (DEPs) in mouse kidneys were filtered followed by standardized analysis workflow (Figures 2A and S1A). Details of quality control were provided in Figures S1B and C. The details about digging out hub genes and pathways related are presented in Figure S1D. In brief, we performed the enrichment analysis using upregulated and downregulated DEGs/DEPs separately and presented functional characteristics accordingly (Figure S1E). After molecular complex detection (MCODE) analysis, we picked out the top five clusters consisting of 334 DEGs followed by CentiScaPe analysis to obtain 49 hub genes (Figure S2A). By matching the top ingenuity pathway analysis (IPA) canonical pathways with hub genes, we found two most significantly enriched genes—*Itgam* and *Itgb2*, that encoded two subunits constituting the heterodimer Mac‐1 (Figure 2B). The key functional role of *Itgam* was also highlighted by IPA canonical pathway and biomarker analysis, where the integrin signaling pathway and *Itgam* ranked at the top, respectively (Figures 2B and S2B, C). In day‐21 group, *Itgam* and ITGAM were upregulated to 19‐fold and 3.3‐fold of their respective levels in the day‐0 group, both at the mRNA and protein level (Figures 2C and D). To investigate the potential regulatory pathways of ITGAM, we identified significant DEGs and DEPs that were positively correlated with Itgam/ITGAM. Subsequently, Kyoto Encyclopedia of Genes and Genomes (KEGG) pathway enrichment analysis was conducted (Figure 2E). Gene Set Enrichment Analysis (GSEA) analyses were also performed using these DEGs and DEPs, which revealed that the Focal Adhesion pathway ranked highest (Figure 2F). Considering the extensive literature on tyrosine kinases as key signal transducers for integrins,^18^ we hypothesized that ITGAM significantly activated focal adhesion signaling. Collectively, multiple biostatistical analyses identified *Itgam* as a hub gene, with significant upregulation in the day‐21 group compared with the day‐0 group, suggesting its potential role in regulating focal adhesion signaling pathway.

### 2.3 Macrophage *Itgam* is highly expressed with alternative M2 polarization in mice with hyperuricemia‐related CKD

**FIGURE 3:**
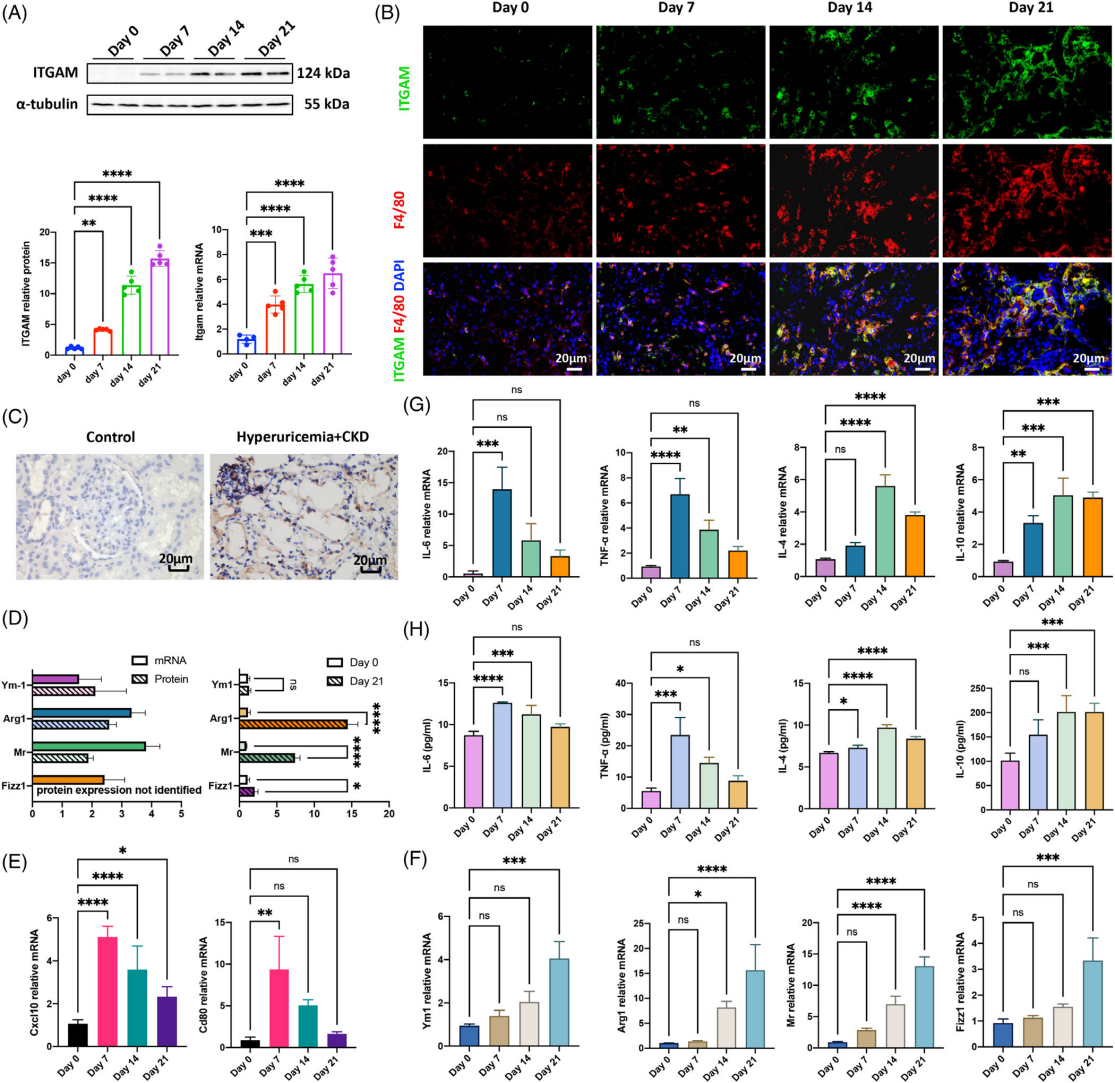
ITGAM expression and macrophage M2 polarization in vivo. (A) ITGAM exhibited increased upregulation in vivo from day 0 to day 21 at both mRNA and protein levels. (B) Expression of ITGAM showed a strong correlation with the macrophage marker F4/80, supported by colocalization of FITC‐labeled ITGAM and TRITC‐labeled F4/80 on the macrophage surface. (C) ITGAM was significantly upregulated in renal biopsy tissue from patients diagnosed with hyperuricemia‐induced CKD compared with human peritumoral kidney tissue. (D) Almost all the M2‐trait genes or proteins (*Arg‐1*/ARG‐1, *Fizz‐1*/FIZZ1, *Mr*/MR, and *Ym1*/YM1) in mice renal tissue were significantly upregulated from the day‐21 group compared with the day‐0 group, as verified by qPCR. (E) Dynamic changes of representative M1‐trait markers (*Cxcl10* and *Cd80*) were examined from day 0 to day 21. (F) Dynamic changes of representative M2‐trait markers (*Arg‐1*, *Fizz‐1*, *Mr*, and *Ym1*) were examined from day 0 to day 21. (G) Classic M1‐trait (*Il6* and *Tnfa*) and M2‐trait (*Il4* and *Il10*) genes were examined from day 0 to day 21 using renal tissue supernate. (H) Classic M1‐trait (IL‐6 and TNF‐α) and M2‐trait (IL‐4 and IL‐10) cytokines were examined from day 0 to day 21 using renal tissue supernate. The sample size for Figure 3A was *n* = 5, and the rest had a sample size of *n* = 3. ****p < 0.0001, ***p < 0.001, **p < 0.01, *p < 0.05.

Based on findings from integrated bioinformatic analysis, we further verified the expression and location of *Itgam* in hyperuricemia‐related CKD. Compared with day 0, *Itgam* expression significantly increased on day 7, 14, and 21 with a trend continuous growth at both of mRNA and protein levels (Figure 3A). Considering *Itgam* mainly but not only expressed in macrophage, we investigated its location in kidney tissue and found it highly colocated with macrophage marker F4/80 in tubulointerstitial and glomerular mesangial space (Figure 3B). Furthermore, upregulation of ITGAM was confirmed by human renal biopsy section in CKD patients with primary hyperuricemia (Figure 3C). Both of transcriptomics and proteomics data revealed activated macrophage M2 polarization in kidney tissue on day 21, clarified by greatly increased mRNA expression of *Arg1* and *Mr* (Figure 3D). We further performed qPCR and compare M1‐trait and M2‐triat membrane markers, and found activation of M1 polarization on day 7, followed by increasingly M2 polarization till day 21 (Figures 3E and F). Similar trends were also observed in M1 and M2 specific cytokines (Figures 3G and H). The evidence presented above, from both animal and human studies, as well as from multiple time points, together suggests that the high expression of *Itgam* is associated with macrophage M2 polarization and is also correlated with hyperuricemia‐mediated renal fibrosis.

### 2.4 UA activated ITGAM and focal adhesion signaling pathway

**FIGURE 4:**
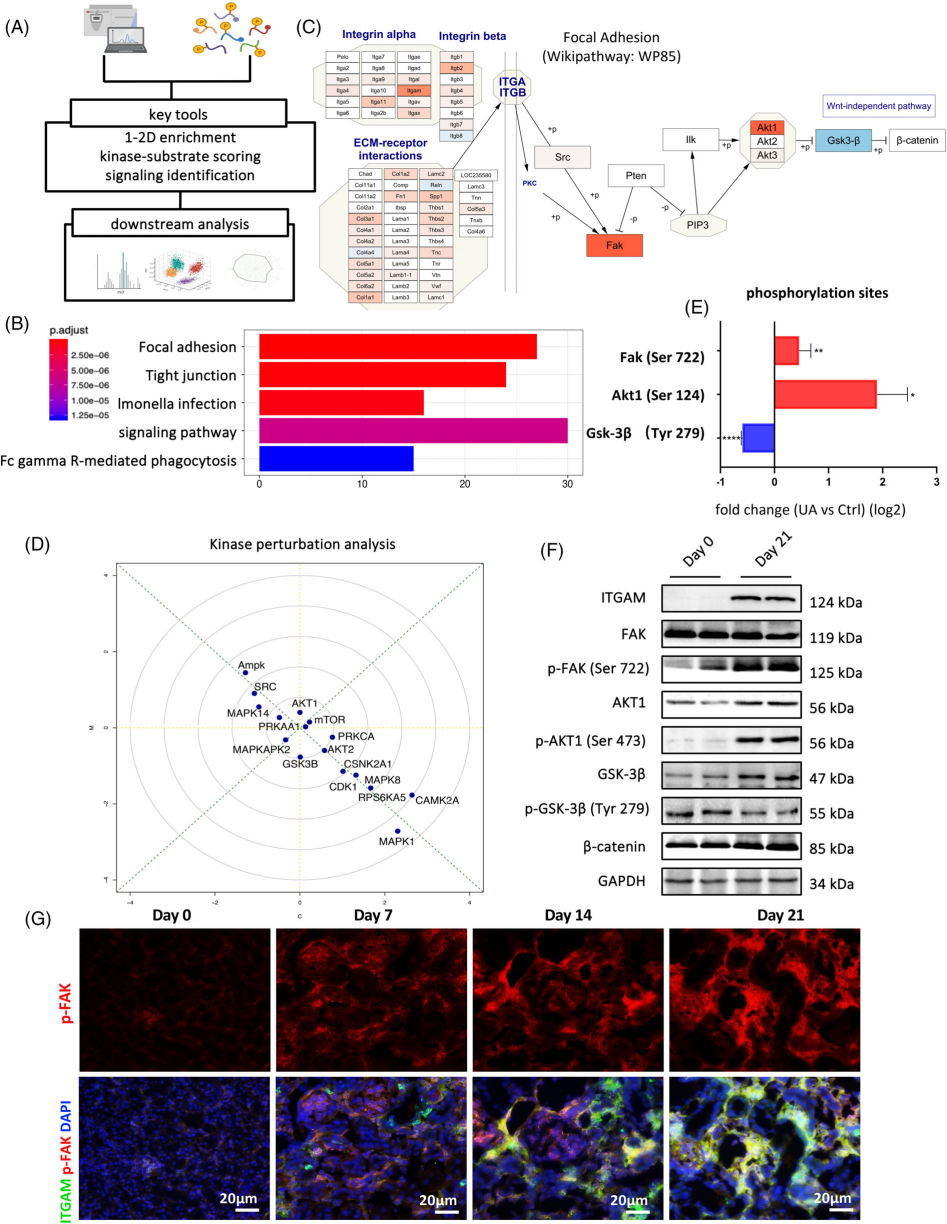
Phosphoproteomics data reveals focal adhesion pathway as essential downstream of ITGAM. (A) The workflow for phosphoproteomics sequencing and statistical analysis was implemented, integrating proteomics and phosphorylation modification data from mice renal tissue in the day‐21 group compared with the day‐0 group. (B and C) KEGG enrichment and pathway analyses both indicated focal adhesion pathway predominantly activated as downstream pathway of ITGAM. (D) Kinase perturbation analysis revealed activation of GSK‐3β and AKT1 were greatly downregulated and upregulated, respectively. (E) Phosphorylation site of enriched molecules in focal adhesion pathway. (F) FAK/AKT1/GSK‐3β pathway was activated in vivo on day 21 versus day 0. (G) ITGAM and p‐FAK expression were highly colocated in the tubulointerstitial compartment.

To reveal disease mechanism from various dimensions, we integrated phosphoproteomics sequencing to further elucidate the main pathway at the protein modification level. Based on results from KEGG enrichment, pathway analysis and kinase perturbation analysis (Figure 4A), we observed focal adhesion pathway (Wikipathway: WP85) was greatly activated by multiple integrins and extracellular matrix components, with phosphorylation of FAK and AKT1 significantly upregulated and GSK‐3β downregulated (Figures 4B–D). Phosphorylation data demonstrated Ser722, Ser124, and Tyr279 were mostly fluctuated in FAK, AKT1, and GSK‐3β, respectively (Figure 4E). These findings were supported by phosphorylation quantification using renal tissue from day 21 compared with day 0 (Figure 4F). Highly colocated p‐FAK and ITGAM indicated the hypothesis that activation of FAK signaling might be related to ITGAM and they coworked together mediating macrophage polarization in hyperuricemia‐related CKD (Figure 4G).

### 2.5 UA directly activated macrophage M2 polarization

**FIGURE 5:**
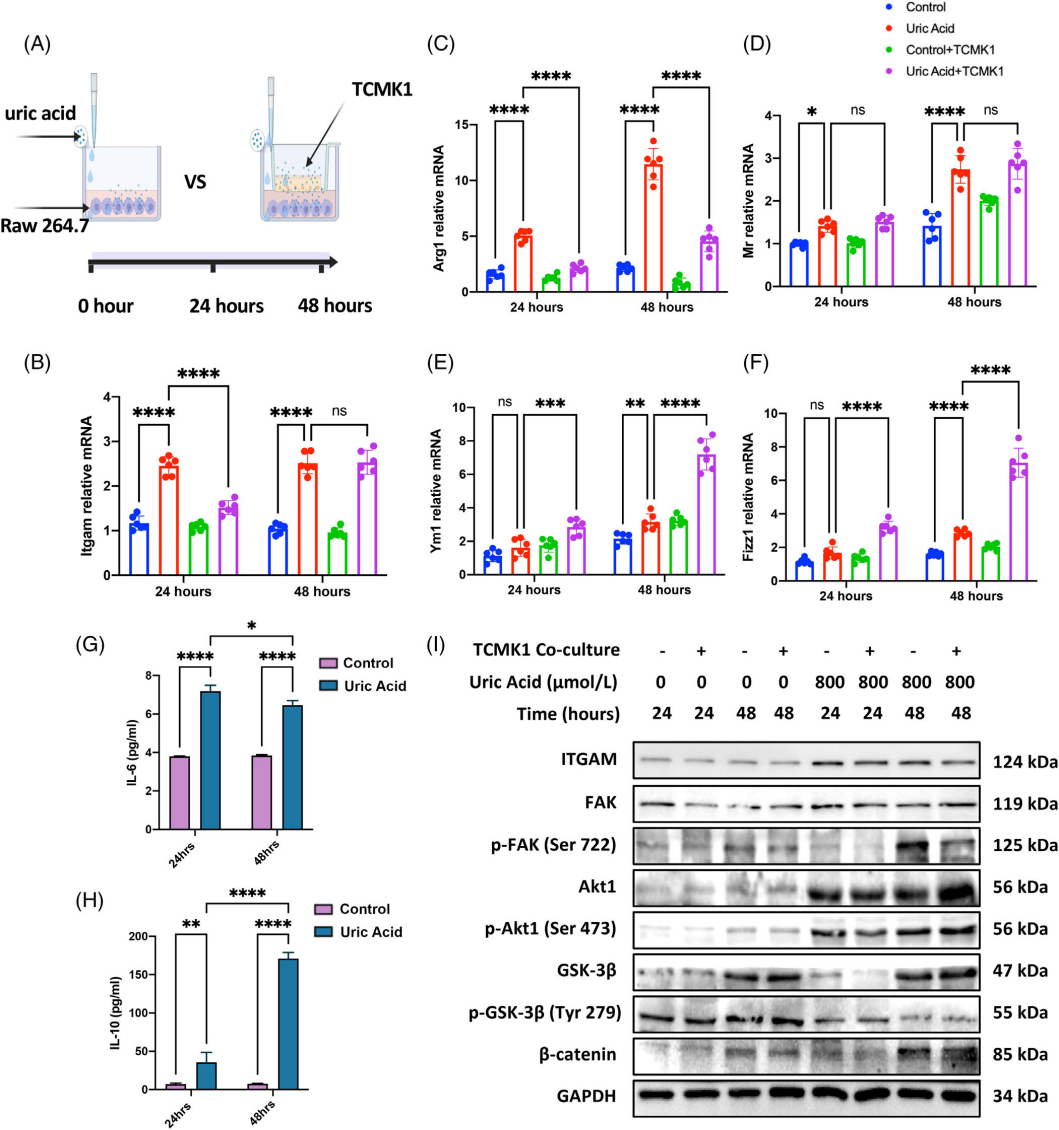
Two experimental models in vitro validated overexpression of ITGAM, M2 polarization of macrophages, and pathway activation. (A) study design of uric acid at 800 µM stimulating Raw 264.7 vs. Raw 264.7 +TCMK1; (B) Raw 264.7 alone, under stimulation of uric acid, greatly overexpressed ITGAM and the expression levels in two models at 48 hours were not significantly different; (C–F) Either Raw 264.7 or co‐culture system presented obvious activation of macrophage M2 activation after uric acid exposure; (G) M1‐trait cytokine IL‐6 in cell culture supernatant was upregulated after 24 hours of uric acid stimulation and remained unchanged thereafter; (H) M2‐trait cytokine IL‐10 continued to rise after uric acid treatment and showed a significant increase from 24 hours onwards; (I) Two models at different time points showed activation of ITGAM/FAK/Akt/β‐catenin pathway. ****P < 0.0001, ***P < 0.001, **P < 0.01, *P < 0.05.

We performed in vitro experiments to explore how UA functions on macrophage. Since UA at high concentration causes tubular damages,^19^, ^20^ we designed two in vitro models: model 1, using UA to stimulate Raw 264.7 macrophage; and model 2, using UA to stimulate the coculture environment of Raw 264.7 + proximal tubular cell TCMK1. Macrophages were collected after 24 and 48 h of stimulation (Figure 5A). *Itgam* mRNA expression in model 1 quickly increased to peak after 24 h, and *Itgam* in model 1 was higher or equal to that in model 2 at time points of 24 and 48 h, respectively (Figure 5B). Although not highly consistence in M2 polarization markers, two models both presented obvious M2 polarization especially after 48 h (Figures 5C–F). Cytokine changes also indicated M1 polarization at early stage and progressively M2 polarization from 24 to 48 h (Figures 5G and H). ITGAM expression and hypothesized downstream FAK signaling were activated since 24 h of UA stimulation. As indicated by KEGG enrichment, we confirmed the higher phosphorylation of FAK and AKT1, lower phosphorylation of GSK‐3β, and less degraded β‐catenin (Figure 5I). Evidence above indicated that UA strongly activated ITGAM/FAK signaling in macrophage and M2 polarization, regardless of existence of tubular epithelial cells.

### 2.6 ITGAM induced macrophage M2 polarization through activating FAK/AKT1/GSK‐3β signaling

**FIGURE 6:**
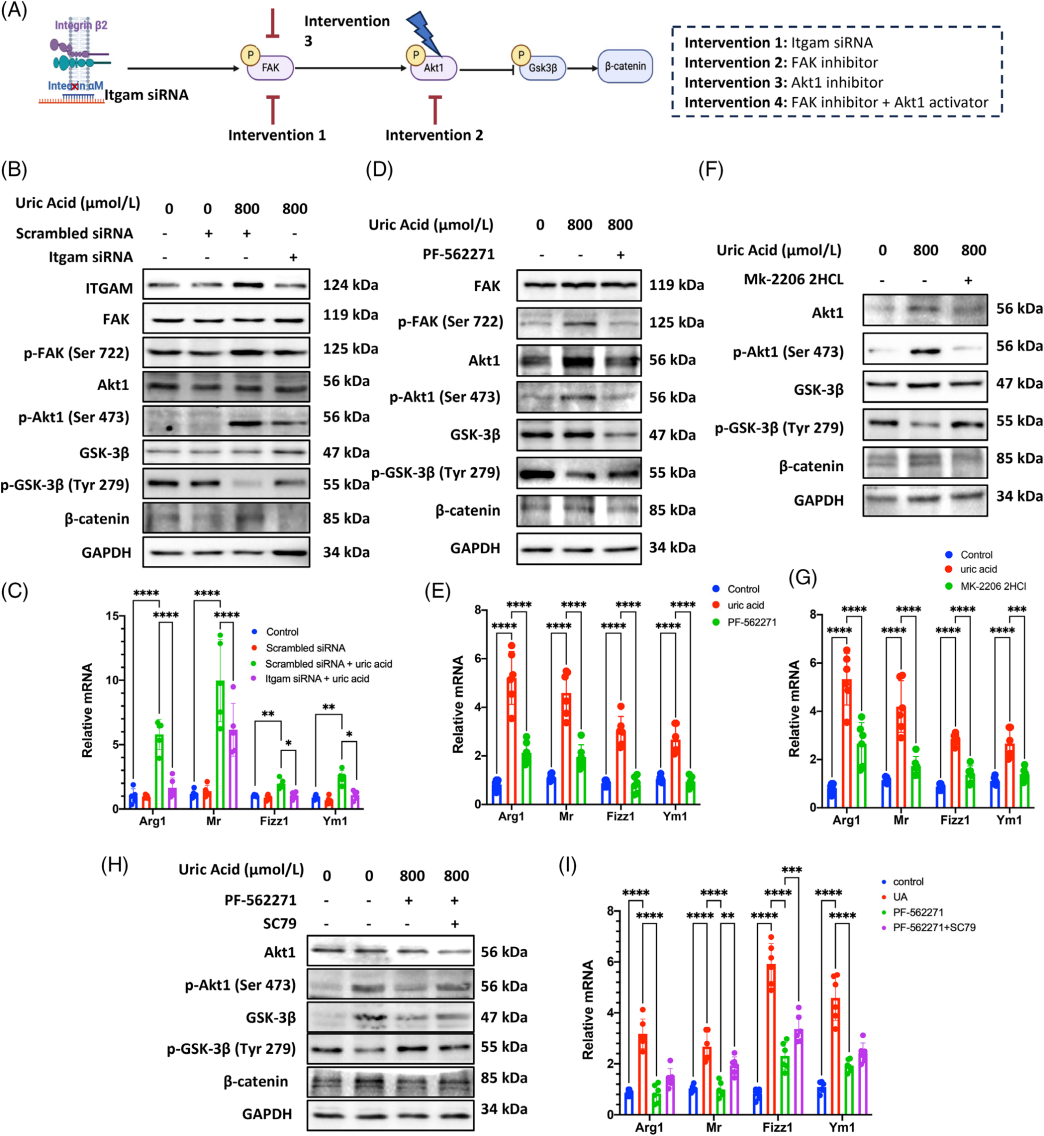
Interventions of ITGAM, FAK, and AKT1 verified the participation of focal adhesion pathway in macrophage M2 polarization in hyperuricemia‐related CKD. (A) Overall design of ITGAM and pathway intervention. (B and C) Silencing of Itgam significantly inhibited phosphorylation of FAK and AKT1, while activating phosphorylation of GSK‐3β and attenuating M2 markers including *Arg1, Mr, Fizz‐1*, and *Ym1*. (D and E) Inhibition of p‐FAK greatly inhibited downstream pathway and macrophage M2 polarization. (F and G) Inhibition of p‐Akt downregulated β‐catenin and macrophage M2 polarization. (H and I) Concurrent inhibition of p‐FAK and activation of p‐AKT1 revealed p‐AKT1 as the downstream of p‐FAK. ****p < 0.0001, ***p < 0.001, **p < 0.01, *p < 0.05.

Based on omics data, we adopted several interventions targeting key molecules in focal adhesion pathway to verify our hypothesis. The mechanism was verified by silencing *Itgam* mRNA, inhibiting p‐FAK, and p‐AKT1 phosphorylation, respectively, followed by combination of inhibiting p‐FAK and activating p‐AKT1 (Figure 6A). As shown by pre‐experiment, we picked the *Itgam* siRNA with the highest knockdown efficiency (mean difference = 77.6%; Figure S3). After knocking down *Itgam*, we observed the decreased phosphorylation of FAK and AKT1, and reversed the upregulation of phosphorylated GSK‐3β, together with downregulation of β‐catenin protein (Figure 6B). Meanwhile, M2 polarization was greatly attenuated after Itgam knockdown, indicated by significant decrease in *Arg1*, *Mr*, *Fizz1*, and *Ym1* mRNA expression (Figure 6C). Similar effects were found after p‐FAK and p‐AKT1 intervention. After inhibiting phosphorylation of these two proteins, the downstream signaling was significantly suppressed together with reduced M2 polarization (Figures 6D–G). The suppression of M2 polarization achieved by inhibiting p‐FAK, was reversed upon activation p‐AKT1, which revealed the downstream involvement of AKT1 regulated by FAK (Figures 6H and I). The consistent result from the intervention experiments above indicates that ITGAM functions through the FAK/AKT1/GSK‐3β signaling within the focal adhesion pathway, leading to the accumulation of β‐catenin and promoting M2 polarization.

## 3 DISCUSSION

Macrophages promotes renal fibrosis and M2 macrophages is strongly associated with kidney fibrosis in both human and experimental diseases. In this study, we report that macrophage ITGAM contributes to renal fibrosis in hyperuricemia‐related CKD. Mechanistically, ITGAM promotes macrophage M2 polarization through activating FAK/AKT1/GSK‐β pathway.

ITGAM, also known as CD11b, was commonly used as a monocyte/macrophage surface marker.^21^, ^22^ Gradually, ITGAM diverse functions were reflected by its rapid confirmational change that alters affinity for its more than 40 ligands, including ICAM‐1,^23^ fibrinogen,^24^ fibronectin,^25^ GPIbα,^26^ RAGE,^27^ JAM‐c,^13^ and others.^28^ We examined the expression of frequently reported main ligands and found a significant upregulation of ICAM‐1 and fibronectin (Figures 1F and S4A, B), which indicated potential role of ITGAM in cell–ECM and cell–cell interactions in kidneys of hyperuricemia‐related CKD. Further studies are needed to clarify how ITGAM mediates crosstalk between macrophage and other cells or compartments in kidney tissue. ITGAM was reported to be involved in various immune responses but with bidirectional effects.^29^ Negative regulation of ITGAM could be observed in systemic lupus erythematous and acute infectious diseases.^30^, ^31^ On the contrary, ITGAM positively regulated chronic inflammatory diseases.^32^ Lange‐Sperandio et al.^13^ reported upregulated Mac‐1 and its ligands ICAM‐1 and JAM‐3 in murine unilateral ureteric obstruction model, and found knockout of Mac‐1 greatly attenuated renal fibrosis. Taking our results together, the functional role of ITGAM in contributing to renal fibrosis through promoting M2 polarization could be inferred.

Each of the α and β subunits of integrins comprises a single transmembrane domain and a short cytoplasmic tail, necessitating interaction with downstream tyrosine kinases to facilitate “outside‐in” signal transduction.^9^, ^33^ Binding to ligands (e.g., extracellular matrix) induces integrins clustering at focal adhesions and connecting to intracellular molecules. FAK as a pivotal mediator in the focal adhesion signaling pathway, is one of the initially identified key elements involving central integrin signaling mechanisms. Once activated, FAK undergoes autophosphorylation, and triggers the activation of multiple downstream effectors associated with diverse signaling pathways. These include SRC, AKT1, Grb7, and others.^34^ Numerous studies reported FAK/AKT1 pathway participated in chronic inflammation and fibrosis, such as atherosclerosis,^35^ and lung and liver fibrosis.^36^, ^37^ Of notes, we additionally observed the change in SRC phosphorylation and found p‐SRC were both attenuated after silencing Itgam and inhibiting p‐FAK. Our finding indicated that SRC participated in ITGAM/FAK signaling and might act as the downstream molecular. It is to be further explored whether SRC together with FAK stimulates AKT1, or acts through other pathways, such as STAT3 and ERK.^38^, ^39^, ^40^ β‐Catenin activation, as the key component of Wnt signaling, is associated with macrophage M2 polarization in tumor malignant and renal fibrosis.^17^, ^41^ Multiple studies have demonstrated that the activation of AKT1 and subsequently decreased phosphorylation of GSK‐3β result in the stabilization of β‐catenin. Accumulated β‐catenin in cytosol then translocates into nucleus and stimulates transcription of target genes related to tissue fibrosis.^42^, ^43^

To our best acknowledgement, this is the first paper investigating the role of integrin and how it regulates macrophage M2 polarization in kidneys of hyperuricemia‐related CKD. Hyperuricemia‐related, especially hyperuricemia‐induced CKD is relatively less studied, due to long time and difficulty when modeling. We firstly fed mice with potassium oxonate only at high dose (4.8 g/kg) for up to 14 weeks with the purpose of establishing UA‐induced CKD, but only found moderately increased serum UA (1.5–2 times of baseline) and almost unchanged kidney tissue histology. Urate oxidase‐knockout mouse is an optimal option, but low survival greatly limited experiment feasibility and reproducibility.^44^, ^45^ Not only signal pathway ITGAM regulates, how ITGAM mediates cell–cell or ECM–cell crosstalk is also potential mechanism of great meaning to be further explored.

In summary, macrophage integrin ITGAM might play the vital role in mediating kidney fibrosis of hyperuricemia‐related CKD through activating its downstream focal adhesion pathway and promoting macrophage M2 polarization. This finding provides new insight into the prevention and treatment of kidney fibrosis and acts as potential therapeutic target, although more well‐designed studies are needed to verify further mechanisms.

## 4 METHODS

### 4.1 Animals

Seven‐week male mice (C57BL/6J, 22−25 g) were purchased from Dossy Experimental Animal Co., Ltd (Chengdu, China). These mice were maintained in the controlled environment for 1 week (temperature at 20 ± 2°C, humidity at 50−60%, and 12‐h light/dark cycle) and provided with food and water ad libitum. To study dynamic characteristic of UA‐induced kidney injury, we randomly divided mice into four groups: day 0, day 7, day 14, and day 21 groups (*n* = 6 per group). The mice in group day 7, 14, and 21 were administered with a mixture of adenine (0.16 g/kg) and potassium oxonate (2.4 g/kg) by oral gavage once daily, as previously described by our team.^38^, ^46^ At day 0, 7, 14, and 21, each group anesthetized with pentobarbital sodium (50 mg/kg, i.p.) and sacrificed. Terminal blood samples were immediately centrifuged and stored at −80°C after collection. Kidney was divided and samples were processed and kept in 10% phosphate buffered formalin, 2.5% glutaraldehyde, or liquid nitrogen depending on purpose, and followed by quick delivery to storage. Animal research was performed in strict accordance with the Animal Research: Reporting of In Vivo Experiments guidelines.^47^

### 4.2 Biochemical measurements

We used fully clinical chemistry analyzer (Mindray BS‐240) to test all the biochemical measurements as previously reported,^48^ including: serum uric acid (µmol/L), SCr (µmol/L), blood urea (mmol/L), alanine aminotransferase (U/L), aspartate aminotransferase (U/L), total cholesterol (mmol/L), serum triglycerides (mmol/L), urine albumin (g/L), and urine creatinine (µmol/L).

### 4.3 Transcriptomics

The mRNA was sequenced by Majorbio Bio‐Pharm Technology Co. Ltd. (Shanghai, China). To summarize, we extracted total RNA using TRIzol® Reagent (Invitrogen, CA) and then checked the samples for integrity, quality, and purity. Next, double‐stranded cDNA synthesis was carried out using a SuperScript double‐stranded cDNA synthesis kit (Invitrogen, CA) with random primers from Illumina. Once we measured and selected the right size, we sequenced the mRNA using an Illumina sequencer, resulting in 2 × 150 bp reads. Finally, we aligned these reads to a reference genome, creating a clean dataset ready for bioinformatic analysis.

### 4.4 Proteomics and phosphoproteomics

Protein was sequenced by PTM‐Biolab Co. Ltd (Shanghai, China). Kidney tissue containing 30 mg of protein was digested with trypsin. After digestion, eluates were desalted by Strata X C18 SPE column (Phenomenex) and vacuum‐dried. TMT labeling was performed according to the manufacturer's protocol for TMT kit/iTRAQ kit. The tryptic peptides were fractionated by high pH reverse‐phase HPLC using Thermo Betasil C18 column (5 µm particles, 10 mm ID, 250 mm length). Samples were dried down and were stored at −20°C before LC–MS/MS measurement.

We prepared our samples with 0.1% formic acid and analyzed them using a special setup that combines ultra‐performance liquid chromatography with a Q Exactive™ Plus mass spectrometer from Thermo Fisher. An electrospray voltage of 2.0 kV was applied. The mass‐to‐charge ratio (*m*/*z*) scan range from 350 to 1800 under the full scan mode, where intact peptides were identified in the Orbitrap detector with a resolution of 70,000. For MS/MS, peptides were selected with a normalized collision energy setting of 28, and the resulting fragments were analyzed in the Orbitrap at a resolution of 17,500. The analysis followed a data‐dependent procedure that alternated between one MS scan and 20 MS/MS scans, incorporating a 15.0‐s dynamic exclusion policy. We controlled the intensity of the ions we were measuring to not exceed 50,000 (automatic gain control), and started measuring at a *m*/*z* of 100.

MS/MS data were identified by matching the raw data to the UniProtKB mouse database (version v06.06.14) using MaxQuant version 1.5.2.8 and its built‐in Andromeda search engine for peak detection and quantification. Search parameters were set as follows: (i) full tryptic specificity was up to four missing cleavages; (ii) mass tolerance for precursor ions was 20 ppm in First search and 5 ppm in Main search; (iii) mass tolerance for fragment ions was 0.02 Da; (iv) carbamidomethyl on cysteine was set as fixed modification; and (v) acetylation modification and oxidation on Met were specified as variable modifications. FDR was adjusted to <1% and minimum score for modified peptides was set >40.

### 4.5 Bioinformatic analysis

All the omics datasets were based on unified gene symbol. For proteomic data, the highest interquartile range of transcript or peptide was chosen if multiple of them were mapped to the same gene. DEGs and DEPs were defined as FDR < 0.05 and absolute value of fold change > 1.5. The DEG and DEP dataset for further analysis consisted of those genes differentially expressed on mRNA and protein levels, respectively. Gene ontology, KEGG pathway enrichment analyses, and IPA were subsequently conducted to identify biological characteristics, biomarkers, and pathways related. We used MCODE plugin from Cytoscape to analyze top clusters from the network of interactions between DEGs. We picked out the top 5 clusters and performed CentiScaPe to calculate centrality indexes and identify hub genes. Canonical pathway analysis in IPA led pathways based on these genes. Target gene was decided after comprehensively incorporating pathways and hub genes enriched. Finally, we selected DEGs positively correlated with targes gene and investigate potential pathways.

### 4.6 Kidney histology

Kidney samples were dehydrated with 10% phosphate buffered formalin followed by paraffin‐embedding. Tissue sections of 4 µm were stained with PAS and Masson trichrome stain for morphology and fibrosis assessment, respectively. The experimental procedures for PAS staining and Masson staining were performed as previously reported.^49^, ^50^ These sections were viewed using light microscopy and were semi‐quantitatively estimated for renal tubular damage according to following scoring system: 0 for 0%, 0.5 for <10%, 1 for 10−25%, 2 for 26−50%, 3 for 51−75%, and 4 for 76−100%. Severity of renal interstitial fibrosis was estimated according to the following four grades: 0% fibrosis (grade 0); 1−25% fibrosis (grade 1); 26−50% fibrosis (grade 2); and >50% fibrosis (grade 3) in interstitial area.^51^

### 4.7 Western blot analysis

Total protein extracted from kidney tissue was loaded into 10−12% SDS‐PAGE gels and then transferred to polyvinylidene difluoride membranes (0.2 µm, Bio‐Rad Laboratories, Inc., Hercules, CA, USA), as previously described. Immunoblots were visualized using and quantified by ImageJ software. Primary antibodies included: α‐tubulin (Abcam; ab179484, 1:1000, RRID: AB_2890906), GAPDH (Zen BioScience; 200306−7E4, 1:5000, RRID: AB_2722713), collagen‐I (Abcam; ab270993, 1:1000), collagen‐IV (Abcam; ab6586, 1:1000, RRID: AB_305584), fibronectin (Boster Biological Technology; ba1772, 1:1000), α‐SMA (Abcam; ab7817, 1:1000, RRID: AB_262054), TGF‐β (Abcam; ab92486, 1:1000, RRID: AB_10562492), IL‐1β (Abcam; ab9722, 1:1000, RRID: AB_308765), IL‐6 (Huabio; EM170414, 1:1000), TNF‐α (Huabio; R1203‐1, 1:1000), ITGAM (Abcam; ab8878, 1:1000, RRID: AB_306831), SRC (Huabio; ET1602‐03, 1:1000), p‐SRC (Huabio; ET1609‐15, 1:1000), FAK (Huabio; ET1602‐25, 1:1000), p‐FAK (Huabio; RT1216, 1:1000), Akt1 (Huabio; ET1609‐47, 1:1000), p‐Akt1 (Huabio; ET1701‐36, 1:1000), GSK‐3β (Huabio; ET1607‐71, 1:1000), p‐GSK‐3β (Huabio; ET1607‐54, 1:1000), β‐catenin (Huabio; EM0306, 1:1000). The density quantitative analysis was conducted by Image J software.

### 4.8 Immunofluorescence staining

Kidney tissue specimens were embedded in O.C.T. compound medium (Tissue‐Tek) and subsequently preserved at a temperature of −80°C. These preserved samples were sectioned into slices of 4‐µm thickness, followed by immediate fixation in 10% PBS‐buffered formalin to maintain tissue integrity, and a sequence of washing and dehydration procedures to prepare them for further histological examination. Sections were blocked with 10% PBS‐buffered horse serum for 1 h at room temperature, incubated with diluted primary antibodies at 4°C overnight, and exposure to second antibodies and DAPI (1:500; Zhongshan Golden Bridge Biotechnology, Beijing, China). Primary and secondary antibodies included: F4/80 (Huabio; RT1212, 1:500), ITGAM (Abcam; ab8878, 1:500), p‐FAK (Huabio; RT1216, 1:250), and TRITC red and FITC green (1:400; Jackson ImmunoResearch), Images were captured using A1R MP+ multiphoton confocal microscope (Nikon) and AxioCam HRc digital camera (Carl Zeiss).

### 4.9 Quantitative real‐time PCR analysis

Total RNA was extracted from kidney tissue specimens using the Total RNA Extraction Kit (BioTek, Winooski, VT, USA). Then reverse transcription was conducted in accordance with the manufacturer's protocol of PrimeScript RT Reagent Kit (Takara Bio, Inc., Otsu, Japan). The polymerase chain reaction (PCR) amplification reactions were quantitatively analyzed using iTaq Universal SYBR Green Supermix (Bio‐Rad Laboratories, Inc.) within CFX Connect PCR system (Bio‐Rad). The data were normalized to GAPDH expression and expressed as relative mRNA levels.

### 4.10 Cell culture and treatments

The mouse macrophage cell line (Raw 264.7) and mouse kidney proximal tubular cell line (TCMK1) were obtained from Shanghai Institute of Biochemistry and Cell Biology, Chinese Academy of Sciences. Raw 264.7 (RRID: CVCL_0493) and TCMK1 (RRID: CVCL_2772) cells were cultured in RPMI 1640 and DMEM/F12 media (both were supplemented with 10% FBS), respectively. To investigate whether tubular cell is the essential mediator of effect from UA on macrophage, we cocultured Raw 264.7 and TCMK1 together by putting transwell inserts carrying TCMK1 to six‐well plates with Raw 264.7. Cells were exposure to UA at concentration of 800 µM, as previously reported.^46^ Based on different intervention purposed, before adding UA, cells were treated with Itgam siRNA (Genepharma Co., Ltd., Suzhou, China), FAK inhibitor (PF562271, Selleck Chemicals LLC, USA), and Akt inhibitor (MK‐2206 2HCl, Selleck Chemicals LLC, USA) as instructed.

### 4.11 Statistical analysis

Results are presented as mean ± standard deviation based on independent experiments. Sample sizes estimation per group were calculated using “resource equation” and confirmed or confirmed by post hoc analyses in G*power 3 software, as previous described. We choose the Student's *t*‐test for two‐group analysis. For comparison of more than two groups, the data were subjected to one‐way analysis of variance, with subsequent pairwise comparisons via the Tukey post‐hoc test. Statistical significance was set at a two‐sided *p* value of less than 0.05.

### 4.12 Materials

Details of all the reagents were listed where they appeared in text for the first time. Primer details are provided in Table S1.

## AUTHOR CONTRIBUTIONS

J. L., L. M., and P. F. conceived and designed the experiments. J. L., F. G., X.‐T. C., and L. M. performed experiments. J. L. and L. M. performed the statistical analyses. J. L., F. G., and L. M. wrote the initial draft of the manuscript. L. M. revised the manuscript. All authors have critically reviewed and revised this paper and approved its final version to be submitted.

## CONFLICT OF INTEREST STATEMENT

The authors declared no conflict of interest.

## ETHICS STATEMENT

This study received approval granted by Animal Care and Use Ethics Committee of Sichuan University (Approval No. 20220303052).

## Supporting information

Supporting Information

## Data Availability

The transcriptomics data are available in the GEO database under accession number “GSE262687”. The mass spectrometry proteomics data have been deposited to the ProteomeXchange Consortium (https://proteomecentral.proteomexchange.org) via the iProX partner repository with the dataset identifier “PXD051046.” Other data related may be obtained from the Supplementary Material or from the corresponding author upon request.
