# Supplementary material for: Integrin αM promotes macrophage alternative M2 polarization in hyperuricemia‐related chronic kidney disease
Source: MedComm (2020). 2024 Jun 22;5(7):e580. doi: 10.1002/mco2.580 (PMC11193137; doi:10.1002/mco2.580)
Supplement: Supplementary file 1 — Supporting Information [file MCO2-5-e580-s001.pdf]

Integrin αM promotes macrophage alternative M2 polarization in hyperuricemia-related chronic kidney disease

Jing Liu [a], Fan Guo [a], Xiaoting Chen [b], Ping Fu [a, *], Liang Ma [a, *]

[a] Division of Nephrology, Institute of Kidney Diseases, West China Hospital of Sichuan University, Chengdu 610041, China.

[b] Animal Experimental Center, West China Hospital of Sichuan University, Chengdu 610041, China

***Correspondence**: Liang Ma and Ping Fu, Division of Nephrology, Institute of Kidney Diseases, West China Hospital of Sichuan University, Guoxue alley 37#, Wuhou District, Chengdu 610041, China. Email: fupinghx@scu.edu.cn (P Fu), liang_m@scu.edu.cn (L Ma).

Running Title: Macrophage ITGAM in hyperuricemia-related CKD

Data availability: The transcriptomics data are available in the GEO database under accession number "GSE262687". The mass spectrometry proteomics data have been deposited to the ProteomeXchange Consortium (https://proteomecentral.proteomexchange.org) via the iProX partner repository with the dataset identifier “PXD051046”. Other data related may be obtained from the Supplementary Material or from the corresponding author upon request.

Funding information: This study was supported by National Natural Science Foundation of China [82300814 and 82370723], and the Science & Technology Department of Sichuan Province [2021YFQ0027 and 2022YFS0589].

Conflict of interest statement: None

Ethics approval statement: This study received approval granted by Animal Care and Use Ethics Committee of Sichuan University (Approval No. 20220303052).

Author contribution statement: J.L., L.M., and P.F. conceived and designed the experiments. J.L, F.G, X-T.C., and L.M. performed experiments. J.L. and L.M. performed the statistical analyses. J.L., F.G., and L.M. wrote the initial draft of the manuscript. L.M. revised the manuscript. All authors have critically reviewed and revised this paper and approved its final version to be submitted.

Supplementary Materials

Supplementary Table1 Detailed information about all the primers used in this research.

Supplementary Figure 1 Differentially expressed gene (DEG) selection and enrichment analysis. (A) DEG selection criteria and details of up-regulated and down-regulated DEGs; (B-C) Quality control results of omics data; (D) Logistics and approach details in selecting target genes and pathways; (E) GO enrichment analysis based on DEGs.

Supplementary Figure 2 Hug gene selection and biomarker analysis. (A) Top 5 clusters of 334 DEGs after MCODE analysis in Cytoscape; (B-C) Incorporating biomarker analysis in IPA and CytoHubba hug gene selection showed ITGAM ranked the 1st at both of mRNA and protein levels.

`

Supplementary Figure 3 Details of Itgam siRNA efficiency and selection.

Supplementary Figure 4 Ligands of ITGAM *in vitro*. (A) qPCR results indicated Icam-1 and Rage two ligands were upregulated at mRNA level; (B) Western blotting showed only ICAM-1 was upregulated.

Supplementary Table1 Detailed information about the primers in this research.

Gens names (species: mouse)	Primers	
	Forward (5’→ 3’)	Reverse (5’→ 3’)
**Gapdh**	CCCCCAATGTATCCGTTGTG	TAGCCCAGGATGCCCTTAGT
**Itgam**	GGAACCAGTGTGGTTGTTGC	GAGGTACTTGCAGGGGGATG
**Icam-1**	CACGTGCTGTATGGTCCTCG	TAGGAGATGGGTTCCCCCAG
**Rage**	GGCATTCAGCTGTTGGTTGAG	CCTGATGCTGACAGGAGGGC
**Gp1ba**	TCCTCCAAAGGACTGTCTGTTC	GCTGTGGAGAAGGTACCCAG
**Jam3**	TGACACGATCGGATTCAGCC	TTCTGGAACCTGGGATTGGC
**Fizz1**	CAGCTGATGGTCCCAGTGAAT	CAGTGGAGGGATAGTTAGCTGG
**Ym1**	GAAGCTCTCCAGAAGCAATCCT	AGAAGAATTGCCAGACCTGTGA
**Arg1**	GTAGACCCTGGGGAACACTAT	CTTCCTTCCCAGCAGGTAGC
**Mr**	GGCCAAGGTACTTCCAGGATT	CCCTGGCACAGCTCATACAT
**Cxcl10**	CCAAGTGCTGCCGTCATTTTC	GGCTCGCAGGGATGATTTCAA
**Il10**	GCTGGACAACATACTGCTAACC	ATTTCCGATAAGGCTTGGCAA
**Tnfa**	CCCTCACACTCAGATCATCTTCT	GCTACGACGTGGGCTACAG
**Il4**	GGTCTCAACCCCCAGCTAGT	GCCGATGATCTCTCTCAAGTGAT
**Il6**	CGGCCTTCCCTACTTCACAA	TTGCCATTGCACAACTCTTTTC
**Il1b**	TGCCACCTTTTGACAGTGATG	AAGGTCCACGGGAAAGACAC

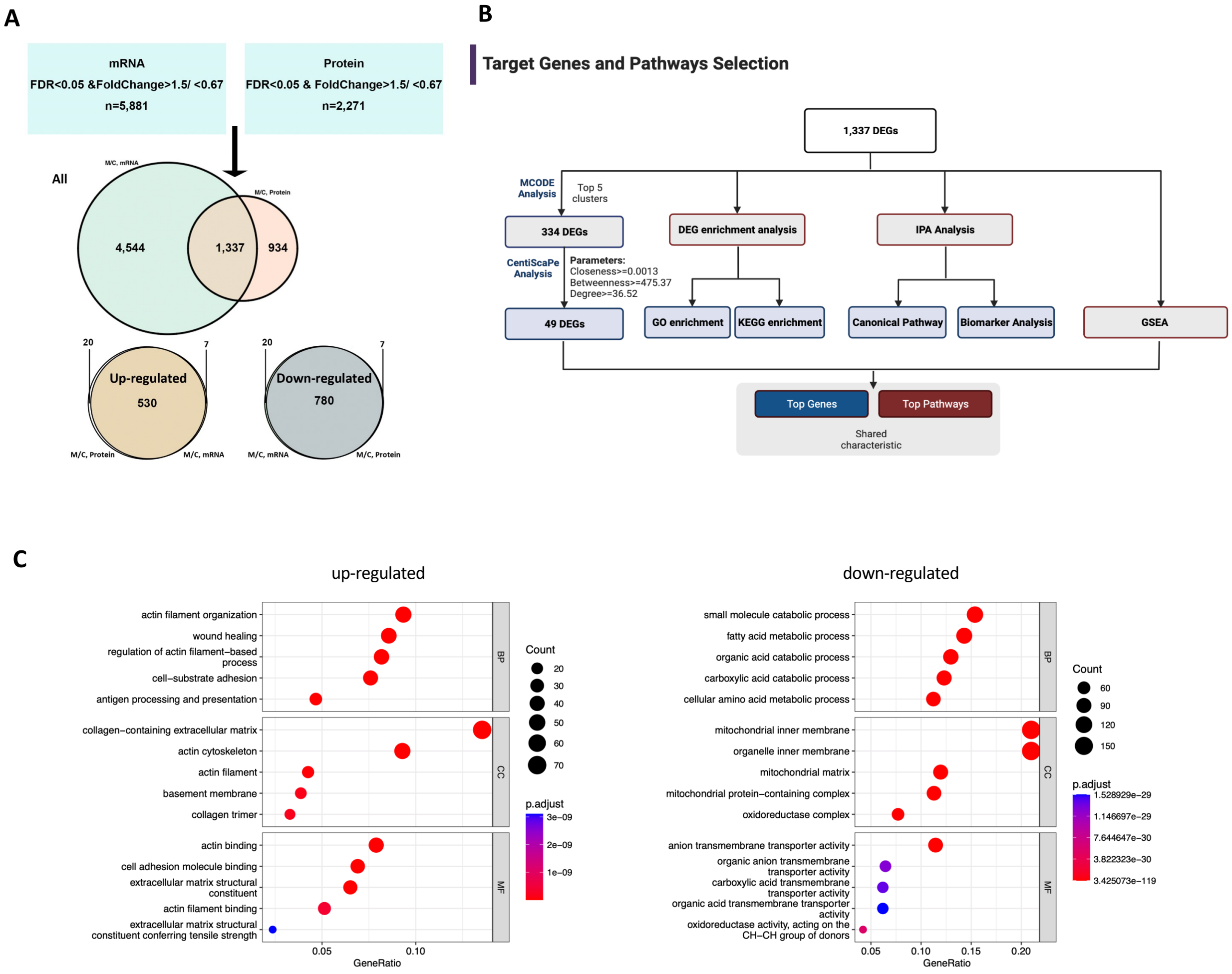

Supplementary Figure 1 Differentially expressed gene (DEG) selection and enrichment analysis. (A) DEG selection criteria and details of up-regulated and down-regulated DEGs; (B-C) Quality control results of omics data; (D) Logistics and approach details in selecting target genes and pathways; (E) GO enrichment analysis based on DEGs.

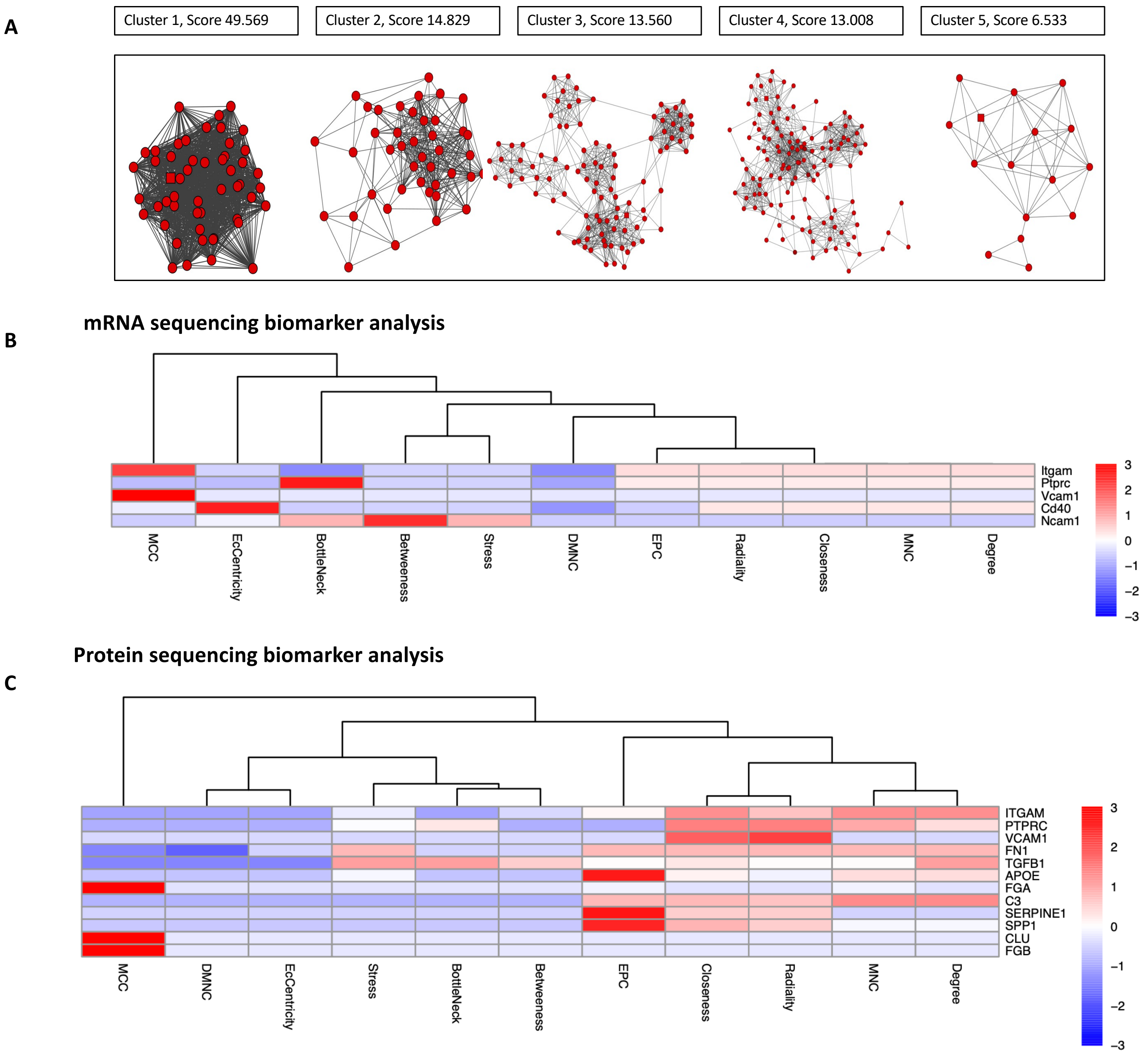

Supplementary Figure 2 Hug gene selection and biomarker analysis. (A) Top 5 clusters of 334 DEGs after MCODE analysis in Cytoscape; (B-C) Incorporating biomarker analysis in IPA and CytoHubba hug gene selection showed ITGAM ranked the 1st at both of mRNA and protein levels.

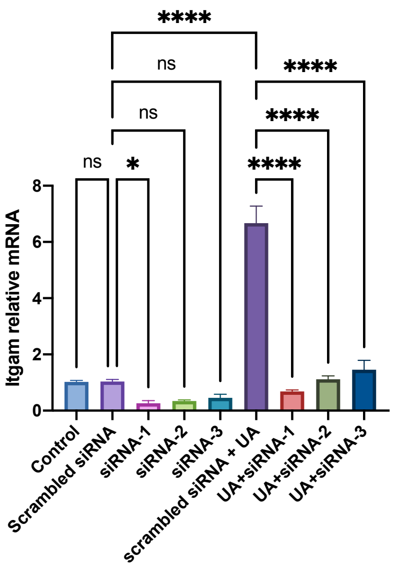

Tukey's multiple comparisons test	Mean Diff.	95.00% CI of diff.	Summary	Adjusted P Value
Scrambled siRNA vs. siRNA-1	0.776	0.08223 to 1.470	*	0.0218
Scrambled siRNA vs. siRNA-2	0.693	-0.0007699 to 1.387	ns	0.0504
Scrambled siRNA vs. siRNA-3	0.5803	-0.1134 to 1.274	ns	0.1454

Supplementary Figure 3 Details of Itgam siRNA efficiency and selection.

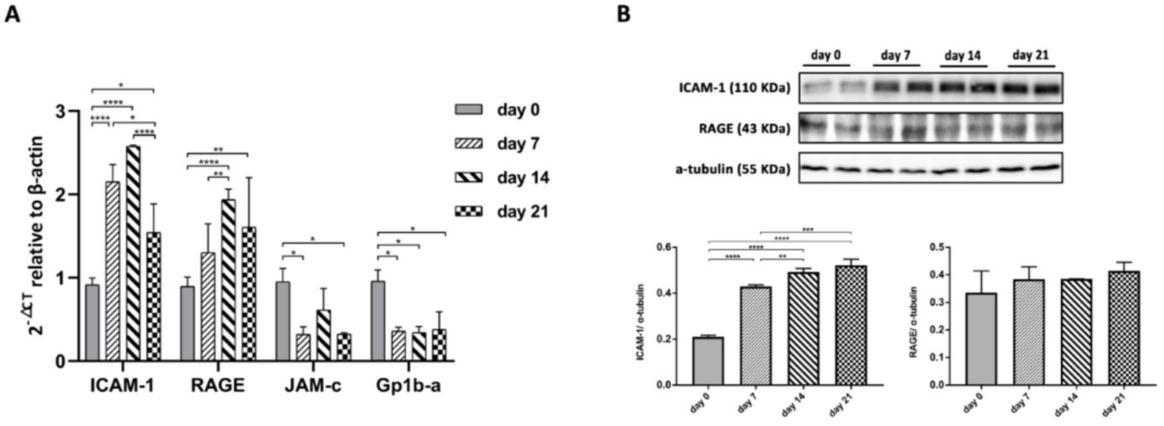

Supplementary Figure 4 Ligands of ITGAM *in vitro*. (A) qPCR results indicated Icam-1 and Rage two ligands were upregulated at mRNA level; (B) Western blotting showed only ICAM-1 was upregulated.
